# Remarkable Psychic Transformation of Mary Vennum

**Published:** 1891-03

**Authors:** 


### REMARKABLE PSYCHIC TRANSFORMATION OF MARY VENNUM,

The case of Mary Vennum, is a strange story. Mary is a young girl, a real flesh and blood heroine, living to-day with her parents in Rollins county, Kan. But in her fourteen years she has lived two lives, two separate, individual existences.

For almost a year this girl lived and talked and ate as an entirely distinct personality. It cannot be said that she *thought* she was this other girl into whose individuality her own had been transferred—she *was* that other girl. The Mary Roff whom she became and remained for nearly twelve months had died several years before. Yet where her life had been broken by death Mary Vennum took it up, continued its interrupted duties, went to live in her own home and could not be dragged away.

She strongly resembled the dead girl, and in pity they let her live in the Roff household, hoping, too, that she would be cured in time, for they thought that she was suffering from a disease.

Her story finally got abroad, and it has puzzled no end of students of such phenomena. Finally Dr. Hodgson, who is the secretary of the English Psychical society, had his attention called to the girl. He has gone carefully step by step over Mary Vennum's whole life, and not only authenticates all the strange details of this tale of transformation, but has gathered much additional material for his society.

Mary was subject to cataleptic fits ; after one of these she didn't know her parents, and began to talk of things about the Roff house and articles in it that her parents knew nothing about. The Vennum family took the girl to the Roff house, as she was always pleading to be taken *home*.

There she stayed perfectly content. From the moment she stepped inside the door she treated all the members of the household as old acquaintances. She understood all their peculiarities as if she had been reared among them. She was perfectly familiar with every piece of furniture and every chair and picture, and seemed in every way happy and contented.

Though she had never even visited the place before, she immediately recognized every object that had belonged to the dead girl, and called it her own. One day she ran through the house several times as though looking for something, and she afterward said to Mrs. Roff : "Mother, where is Gyp? I want to see him. I am afraid he has not been properly cared for."

Gyp had been the favorite pet of Mary Roff, and had been buried about eleven years. His name had never been mentioned before Mary, and the Roffs never remember to have spoken of him since their acqaintance with the Vennums.

Very many instances of a similar nature are told of Mary and fully corroborated by members of the two families.

